# STAT6 Upregulates NRP1 Expression in Endothelial Cells and Promotes Angiogenesis

**DOI:** 10.3389/fonc.2022.823377

**Published:** 2022-05-05

**Authors:** Peng Gao, Guanghui Ren, Jiangjiu Liang, Ju Liu

**Affiliations:** ^1^Department of Gerontology, The First Affiliated Hospital of Shandong First Medical University & Shandong Provincial Qianfoshan Hospital, Jinan, China; ^2^Institute of Microvascular Medicine, Medical Research Center, The First Affiliated Hospital of Shandong First Medical University & Shandong Provincial Qianfoshan Hospital, Jinan, China; ^3^Shandong Provincial Key Laboratory of Animal Resistant, School of Life Sciences, Shandong Normal University, Jinan, China

**Keywords:** endothelial cell, tumor angiogenesis, STAT6, neuropilin-1, transcriptional regulation

## Abstract

The role of signal transducer and activator of transcription 6 (STAT6) in tumor growth has been widely recognized. However, its effects on the regulation of angiogenesis remain unclear. In this study, we found that STAT6 promoted angiogenesis, possibly by increasing the expression of neuropilin-1 (NRP1) in endothelial cells (ECs). Both STAT6 inhibitor (AS1517499) and STAT6 siRNA reduced EC proliferation, migration, and tube-formation, accompanied by downregulation of NRP1, an angiogenesis regulator. Furthermore, IL-13 induced activation of STAT6 and then increased NRP1 expression in ECs. IL-13-induced EC migration and tube formation were inhibited by NRP1 siRNA. Luciferase assay and chromatin immunoprecipitation assay demonstrated that STAT6 could directly bind to human NRP1 promoter and increase the promoter activity. In tumor xenograft models, inhibition of STAT6 reduced xenograft growth, tumor angiogenesis, and NRP1 expression *in vivo*. Overall, these results clarified the novel mechanism by which STAT6 regulates angiogenesis, and suggested that STAT6 may be a potential target for anti-angiogenesis therapy.

## Introduction

Blood vessels, especially the capillaries, run throughout the body, providing oxygen and nutrients and exchanging cellular and tissue byproducts to maintain normal functioning of tissues (1). However, insufficient vascularization causes impaired healing of fractures and placental deficiency, whereas increased vascularization leads to atherosclerosis, hemangioma, and neoplastic development (2). Aberrant vascularization causes a variety of diseases (3) and hence, reasonable arrangement of blood vessels is a therapeutic strategy, especially for cancer treatment.

Tumor cells are not restricted by the cell cycle and grow faster than normal cells. Therefore, more nutrients are needed for tumor cell growth (4). When the diameter of a tumor is larger than 2 mm, more new vessels are needed to support tumor growth (5). Angiogenesis refers to the formation of new blood vessels from the existing vessels, which are tightly regulated by the balance between pro- and anti-angiogenic molecules. However, the balance is unsettled when existing vessels are insufficient to support tumor overgrowth. An increase of pro-angiogenic molecules’ secretion induced by inflammation and hypoxia is commonly seen in the tumor microenvironment, which induces tumor angiogenesis (6). Angiogenesis provides not only nutrition for tumor growth but also a channel for tumor metastasis in tumorigenesis. Therefore, inhibition of angiogenesis has broad prospects for anti-tumor and other angiogenesis-related disease therapy.

Signal transducer and activator of transcription 6 (STAT6), a member of the STAT family, reportedly participates in inflammation and tumorigenesis by regulating the transformation between M1 and M2 macrophages (7, 8). IL-4 or IL-13 bind to IL-4Rα or IL-13Rα1 on the cell surface, and activate the STAT6 signaling pathway by phosphorylating STAT6 on Tyr-641 (9). Two phosphorylated STAT6 monomers form dimers and translocate into the nucleus to bind certain promoters with the sequence of TTCnnnGAA (“n” can be A, T, G, or C) (10–12). High expression of STAT6 has been detected in many types of cancer, including non-small-cell lung cancer and colorectal cancer (13, 14). STAT6 knockout mice have a higher tolerance to lung cancer metastasis than wild-type mice (15). The mechanism of action of STAT6 in tumorigenesis is being gradually understood, but is still unclear in with respect to tumor angiogenesis.

Transmembrane glycoprotein neuropilin-1 (NRP1), which was first found in neuronal and endothelial cells, is essential for normal embryonic development, axon guidance, and angiogenesis (16). Owing to the lack of enzyme activity, NRP1 acts as a co-receptor of VEGFR2 (kinase insert domain-containing receptor; KDR) (17). VEGF binds to NRP1, which promotes the interaction of NRP1 and KDR, and activates downstream signaling events of VEGF (17). NRP1 was initially thought to enhance VEGF binding to KDR (18). However, NRP1 still promotes tumor angiogenesis, in the absence of VEGFR (19). VEGF is widely recognized as a pro-angiogenic factor for stimulating angiogenesis and blockade of the VEGF signal pathway is an effective anti-tumor therapy (20).

In this study, we found that inhibition of STAT6 activity reduced NRP1 expression, and decreased proliferation, migration, and tube-formation of endothelial cells (ECs). Activation of STAT6 by IL-13 increased NRP1 expression and increased proliferation, migration, and tube-formation of ECs. In addition, STAT6 directly bound to the *NRP1* promoter and increased its transcription activity. In the tumor xenografts model, inhibition of STAT6 activation reduced tumor angiogenesis and NRP1 expression, suggesting the therapeutic potential of STAT6 inhibitors.

## Material and Methods

### Cell Lines and Reagents

Human umbilical vein endothelial cells (HUVEC) were purchased from PromoCell (Heidelberg, Germany), and cultured in Dulbecco’s modified Eagle’s medium (DMEM) (Gibco, Carlsbad, CA, USA) with 10% (v/v) fetal bovine serum (FBS) (Gibco) and antibiotics (100 IU/mL penicillin and 100 mg/mL streptomycin) (Gibco). The A549 cell line was purchased from Cell Resource Center of Life Sciences (Shanghai, China), and cultured in Roswell Park Memorial Institute 1640 medium (RPMI 1640) (Gibco) containing 10% FBS (Gibco). HUVEC were treated with recombinant human IL-13 protein (50 ng/mL) (213-ILB, R&D systems, Minneapolis, MN, USA) and STAT6 inhibitor, AS1517499 (AS) (1 mM) (HY-100614, MCE, NJ, USA).

### Knockdown of STAT6 and NRP1

Small interference RNA of human STAT6 and human NRP1 were purchased from GenePharma (Shanghai, China). The sequences of siRNA are shown in **Table 1**. HUVECs (2.5 × 10^4^) were seeded in six-well plates and cultured overnight. *NRP1* siRNA (2 and 3) (100 nM) or *STAT6* siRNA (100 nM) were transfected using Lipofectamine 2000 (Invitrogen, Carlsbad, CA, USA) according to the manufacturer’s instructions. Subsequent experiments were performed after cells were incubated at 37°C in a humidified atmosphere of 5% CO_2_ or 21% O_2_ for 48 h.

**Table 1:** Sequences of siRNA used in this study.

Primer name	Sequence (5'->3')	
Negative control S	UUCUCCGAACGUGUCACGUTT	
Negative control R	ACGUGACACGUUCGGAGAATT	
Human *NRP1* S	CCAUACCAGAGAAUUAUGATT	1
Human *NRP1* R	UCAUAAUUCUCUGGUAUGGTT	
Human *NRP1* S	CAGCCUUGAAUGCACUUAUTT	2
Human *NRP1* R	AUAAGUGCAUUCAAGGCUGTT	
Human *NRP1* S	GUAUACGGUUGCAAGAUAATT	3
Human *NRP1* R	UUAUCUUGCAACCGUAUACTT	
Human *STAT6* S	AGGAAGAACUCAAGUUUAATT	
Human *STAT6* R	UUAAACUUGAGUUCUUCCUGC	

### Mouse Xenograft Assays

All experimental protocols related to animals were performed in compliance with the guidelines of the Animal Care and Use Committee of the First Affiliated Hospital of Shandong First Medical University. The mouse xenograft was performed as in a previous study (21). Briefly, 10 male BALB/c nude mice (5-6 weeks old), weighing roughly 20 g were housed in a specific pathogen-free animal facility and exposed to a 12-h light/dark cycle. A549 (5×10^6^) with Matrigel (356234, BD Biosciences, Franklin Lakes, NJ, USA) mixed by volume ratio 1:1 was injected into the subcutaneous region (the right flanks) of mice, and tumor sizes were determined using the formula: length × width^2^×0.52. Seven days after implantation, when the tumor size reached a volume of 100 mm^3^, mice were randomly divided into two groups. Administration with AS1517499 (25 mg/kg intratumorally injection, twice a week) was started and lasted for 30 days.

### Correlation of STAT6 and Down-Regulated Genes in Lung Adenocarcinoma

The correlation between STAT6 and angiogenesis genes in LUAD was performed *via* Gene Expression Profiling Interactive Analysis (22) (GEPIA, Zemin Zhang’ Lab, Biomedical Pioneering Innovation Center, Peking University, Beijing, China; http://gepia.cancer-pku.cn/), according to a previous report (21).

### Trypan Blue Staining and Cell Counting

HUVECs were seeded in 10-cm dishes and cultured overnight. AS (1 mM) was added, and the cells were incubated at 37°C in a humidified atmosphere at 5% CO_2_ or 21% O_2_ for 24 h. The cells were then digested with trypsin. Then, 0.4% trypan blue (Beyotime Biotechnology, Shanghai, China) was used to assess cell viability after AS treatment. The ratio of live to dead cells was calculated by a Cell Counting Equipment (Jimbio CL, Leso Technology Co., Ltd, Shandong, China) according to the manufacturer’s instructions.

### MTT

HUVECs (3×10^3^) were seeded in 96-well plates and cultured overnight. AS (1 mM) and/or IL-13 (50 ng/mL) were added, and the cells were incubated for 0 h, 24 h, 48 h, and 72 h at 37°C in a humidified atmosphere. After incubation, 10 μL MTT solutions (5 mg/mL) were added to each well and incubated for 4 h. The colorimetric intensity was analyzed using a 96-well plate reader at a wavelength of 490 nm.

### Scratch Test

HUVECs (1×10^6^) were seeded in a 35 mm^2^ Petri dish and cultured overnight. Two parallel mark lines were made in the bottom of the disc using a marker pen, and cells were scratched with a 1000 μL pipette tip perpendicular to the above-mentioned lines. The culture medium was removed and the cells were washed with phosphate-buffered saline (PBS). Cells were further incubated with serum-free medium, which contained AS (1 mM) and/or IL-13 (50 ng/mL). The scratches were photographed at both 0 and 20 h.

### Tube-Formation Assay

HUVECs were treated with AS (1 mM) and/or IL-13 (50 ng/mL) for 24 h.

Matrigel diluted with endothelial cell basal medium-2 (EBM-2) (lonza, Basel, Switzerland) containing 2% FBS was added to a pre-chilled 96-well plate and incubated at 37°C for 40 min, and then HUVECs (2×10^4^) were seeded in 96-well plates. EBM-2 was added to the cells during the process of the assay. The 96-well plate was incubated at 37°C in a humidified atmosphere for 6 h. Tube-formation was observed using an inverted light microscope (Olympus, Tokyo, Japan). ImageJ software (National Institutes of Health, Bethesda, MD, USA) was used to measure the tube length, and tube-formation was expressed as a percentage of the control group.

### Vectors

The 2000-bp fragments of the *NRP1* promoter were obtained from the Eukaryotic Promoter Database (EPD, https://epd.epfl.ch//index.php). Using the *Xho*I and *Hind*III restriction sites, the *NRP1* promoter was cloned into pGL3-basic (Promega, Madison, WI) (named *NRP1*-promoter), and pRL-TK (Promega) was used as the reference control.

### Dual-Luciferase Reporter Assay

HUVECs (2×10^4^) were seeded in 24-well plates and cultured overnight. *NRP1*-promoter (0.5 μg) was transfected using Lipofectamine 2000 according to the manufacturer’s instructions. After 24 h of transfection, AS (1 μM) and/or IL-13 (50 ng/mL) were added to the media, and the 24-well plate was incubated at 37°C in a humidified atmosphere for an additional 24 h. HUVECs in the 24-well plates were lysed for luciferase assay. Luciferase and Renilla activities were determined by a Luciferase-Renilla assay system (E1980, Promega) on an LB960 luminometer (Berthold, Germany).

### Chromatin Immunoprecipitation Assay

HUVECs were fixed with 1% formaldehyde for 10 min at room temperature, and then 1× glycine solution was used to stop fixing. HUVECs were washed twice with ice-cold PBS containing an EDTA-free protease inhibitor mixture (Roche, Basel, Switzerland) and collected by a cell scraper. Fragmentation of genomic DNA was performed by sonication. Immunoprecipitation was performed using a SimpleChIP^®^ Enzymatic Chromatin IP Kit (Magnetic Beads) (9005, Cell Signaling Technology, Danvers, MA, USA) with antibodies for STAT6 (ab32520, Abcam, Waltham, MA) according to the manufacturer’s instructions. Rabbit IgG was used as a negative control. The primer sequences used are listed in **Table 2**. The PCR products were separated on 2% agarose gel and visualized under ultraviolet light (Protein Sample, Silicon Valley CA, USA).

**Table 2:** Primers used in this study.

Primer name	Sequence (5'->3')	ChIP or qRT-PCR
Human *NRP1* F1	CAGGTGATGACTTCCAGCTCA	qRT-PCR
Human *NRP1* R1	CCCAGTGGCAGAAGGTCTTG	
Human *ACTIN* F1	TTGCCGACAGGATGCAGAA	qRT-PCR
Human *ACTIN* R1	GCCGATCCACACGGAGTACT	
Human *STAT6* F1	CTTTCCGGAGCCACTACAAG	qRT-PCR
Human *STAT6* R1	AGGAAGTGGTTGGTCCCTTT	
Human *NRP1* promoter F1	CTTTCCGGAGCCACTACAAG	ChIP
Human *NRP1* promoter R1	AGGAAGTGGTTGGTCCCTTT	

### Angiogenesis Polymerase Chain Reaction Assay and Quantitative Real-Time PCR

HUVECs (1×10^6^) were seeded in a 35-mm^2^ Petri dish and cultured overnight. AS (1 μM) or IL-13 (50 ng/mL) was used to treat HUVECs for 12 h. Total RNA was extracted by using RNAiso Plus (TaKaRa, Kyoto, Japan), and cDNA was synthesized by using a PrimeScript First Strand cDNA Synthesis Kit (TaKaRa) according to the manufacturer’s instructions. Angiogenesis PCR array plates (Wcgene biotech, Shanghai, China) and qRT-PCR were performed by monitoring an increase in fluorescence of SYBR green dye (Tiangen, Beijing, China) using a CFX96TM Real-Time System (Bio-Rad, Hercules, CA, USA). The relative expression of RNA was calculated using actin as an endogenous internal control. Primer sequences are presented in **Table 2**.

### Western Blotting

HUVECs were washed twice with ice-cold PBS and lysed with cell lysis buffer (Beyotime Biotechnology) containing an EDTA-free protease inhibitor (Roche). Tumor tissue was incubated with ice-cold cell lysis buffer containing protease inhibitor and disrupted with Tissuelyser-24. Protein concentration was quantified using the Pierce™ BCA Protein Assay Kit (Thermo Fisher Scientific, Waltham, MA, USA). Western blotting was performed as previously described (23). Blots were incubated with Pierce ™ ECL Western Blotting Substrate (Thermo Fisher), and detected by AI 680 (General Electric Company, Boston, MA) Antibodies used for western blotting included rabbit anti-phospho-STAT6 (Tyr-641, 1:1000, Cell Signaling Technology); rabbit anti-STAT6 (1:1000, Abcam); rabbit anti-NRP1 (1:1000, Affinity); rabbit anti-NRP1 (1:1000, Abcam); and rabbit anti-Tubulin (1:5000, Proteintech). The secondary antibody was horseradish peroxidase (HRP)-conjugated goat anti-rabbit immunoglobulin G (IgG) (1:8000, Proteintech).

### Immunohistochemistry Staining and Immunofluorescence Staining

Immunohistochemistry staining and immunofluorescence staining were performed as described in a previous report (24). Briefly, the tumors were stripped and fixed in 4% paraformaldehyde at room temperature for 24 h, and then embedded in paraffin for sectioning. Sections were dewaxed and dehydrated using an alcohol gradient, heated, and blocked with hydrogen peroxide at room temperature. For the immunohistochemistry staining, rabbit anti-NRP1 (1:1000, affinity) was used as the primary antibodies. After rinsing with PBS, sections were incubated with a secondary antibody from the MaxVisionTM HRP-Polymer anti-mouse/rabbit IHC Kit (Maixin, China). BX51 microscopic imaging system (Olympus) was used to observe the digitized images, and microvessel density was determined with Image J software (National Institutes of Health, Bethesda, MD, USA) by quantifying NRP1-positive pixels in the digitized images. For immunofluorescence staining, mouse anti-CD31 (1:200, Abcam) and rabbit anti-NRP1 (1:200, Abcam, ab25998) were used as the primary antibodies. Alexa Fluor 488 goat anti-rabbit (1:200; Abcam) and Alexa Fluor 594 donkey anti-mouse secondary antibody (1:200; Abcam) were used as the primary antibodies. DAPI was used to display the nucleus. The sections were photographed using an Olympus LCX100 Imaging System (Olympus).

### Statistical Analyses

All values are presented as mean ± standard error of the mean (SEM). Statistical analysis was performed using GraphPad Prism 9 software (GraphPad). An unpaired Student’s *t*-test was used for analyses between two groups; for three or more groups, one-way analysis of variance (ANOVA) followed by Bonferroni’s *post-hoc* test was used for statistical analysis. *P*<0.05 was considered to indicate statistical significance.

## Results

### Inhibition of STAT6 Reduces Migration, Proliferation, And Tube-Formation of ECs

**Figure 1:**
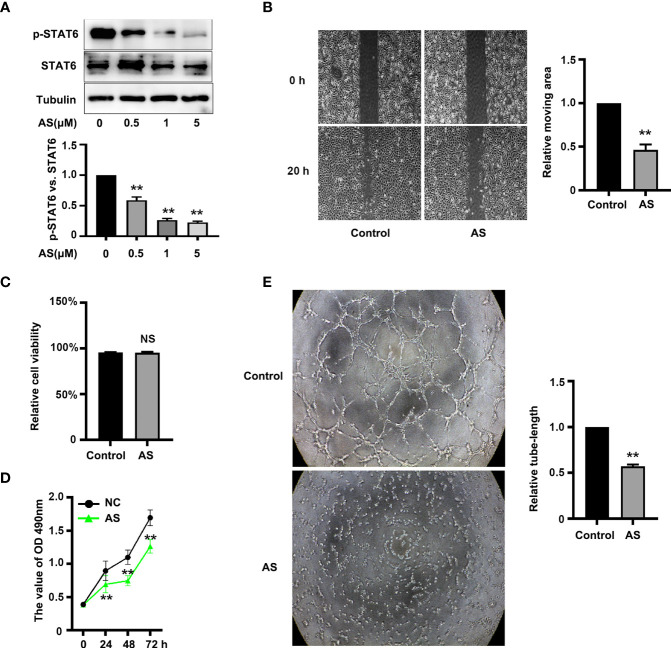
AS treatment reduces STAT6 activity and inhibits HUVEC migration, proliferation, and tube-formation. **(A)** Phosphorylation of STAT6 was detected by western blotting in HUVECs treated with 0.5, 1, or 5 μM AS for 24 h, n=3, *P<0.05 vs. control. **(B)** The typical images and the relative moving area of HUVECs treated with AS (1 μM) for 20 h, n=5, **P<0.01 vs. control. **(C)** The relative cell viability of HUVECs treated with AS (1 μM) for 24 (h.) NS denotes no significant difference, **(D)** The cell growth curve of HUVECs treated with AS (1 μM) for 0 h, 24 h, 48 h, and 72 h, n=5, **P<0.01 vs. control. **(E)** The typical images of STAT6 affected HUVECs tube-formation. HUVECs were treated with AS (1 μM) for 24 h, n=5, **P<0.01 vs. control.

To assess the effect of STAT6 on EC angiogenesis, HUVECs were incubated with different concentrations of STAT6 inhibitor, AS1517499 (AS). The expression of STAT6 was not affected by AS; however, as expected, AS reduced the phosphorylation of STAT6 in a dose-dependent manner (**Figure 1A**). Incubation with 1 μM AS significantly inhibited EC migration (**Figure 1B**), cell proliferation (**Figure 1D**), and tube-formation (**Figure 1E**). Moreover, incubation with 1 μM AS did not increase cell death as compared with the control group (**Figure 1C**). STAT6 siRNA was also used to evaluate the effect of STAT6 on EC angiogenesis. Knockdown of STAT6 significantly reduced STAT6 expression at both the protein and RNA level (**Figures S1A–C**). Proliferation, tube-formation, and migration of ECs were all inhibited by STAT6 expression blockade (**Figures S1D–H**). These results suggest that inhibition of STAT6 with AS reduces EC angiogenesis.

### STAT6 Regulates the Expression of Angiogenic Genes

**Figure 2:**
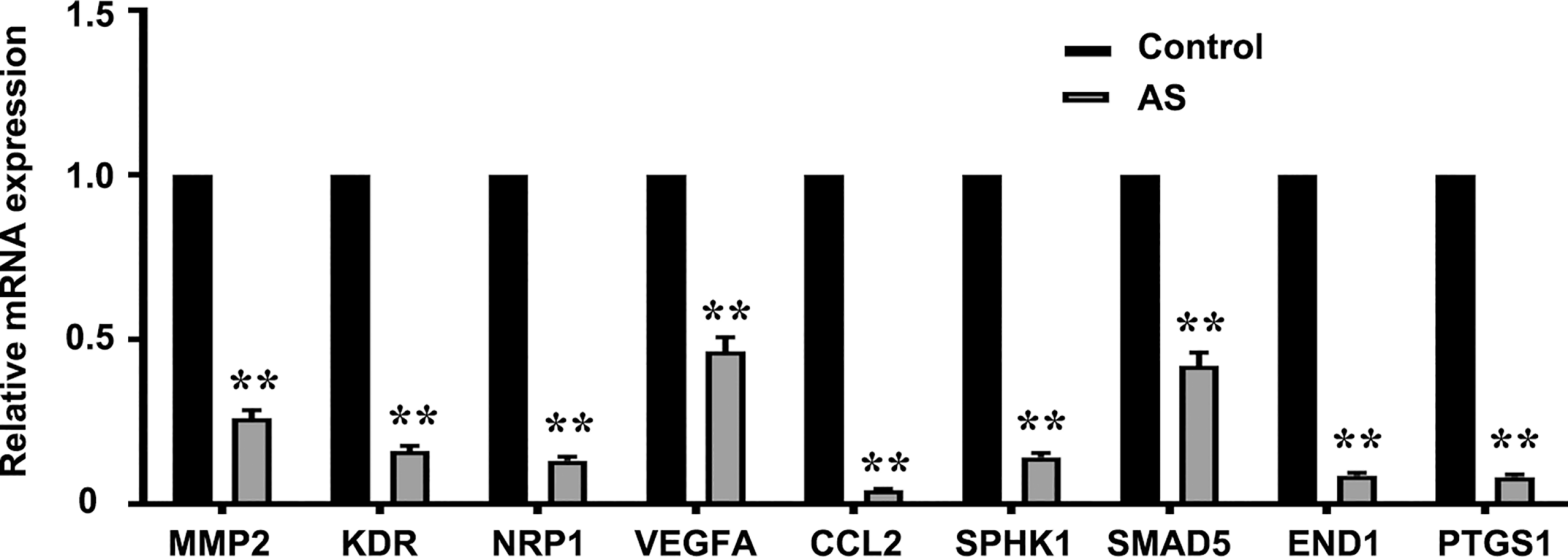
AS treatment reduces pro-angiogenesis gene expression. Pro-angiogenesis genes were detected by PCR in HUVECs treated with an inhibitor of STAT6 (1 μM) for 24 h. **P<0.01 vs. control. MMP2, matrix metalloproteinase-2; KDR, kinase insert domain-containing receptor; NRP1, neuropilin-1; VEGFA, vascular endothelial growth factor A; CCL2/MCP-1, monocyte chemoattractant protein-1; SPHK1, sphingosine kinase 1; SMAD5, mothers against DPP homolog 5; EDN1, endothelin-1; PTGS1/COX1, cyclooxygenase 1.

PCR arrays were used to analyze the expression of pro-angiogenesis genes in HUVECs incubated with 1 μM AS for 24 h. Eighty-six angiogenesis-related genes were detected *via* the qPCR array. Nine genes, *NRP1, MMP2, KDR, VEGFA, CCL2, SPHK1, SMAD5, EDN1*, and *PTGS1*, were significantly down-regulated by AS treatment (**Figure 2**). To further confirm our results, the correlation of mRNA level in LUAD between STAT6 and these genes was analyzed by GEPIA. The analysis was based on the bulk gene expression datasets in the TCGA and the Genotype-Tissue Expression (GTEx) projects. The results of the scatter plot analysis indicated a positive correlation between the mRNA levels of *STAT6* and *NRP1* (R=0.41), *STAT6* and *MMP2* (R=0.39), *STAT6* and *KDR* (R=0.29), *STAT6* and *VEGFA* (R=0.2), *STAT6* and *CCL2* (R=0.23), *STAT6* and *SPHK1* (R=0.36), *STAT6* and *SMAD5* (R=0.2), *STAT6* and *EDN1* (R=0.25), and *STAT6* and *PTGS1* (R=0.27) (**Figure S2**). These data suggest that STAT6 regulates lung tumor angiogenesis by mediating the expression of pro-angiogenic genes.

### STAT6 Affects the Expression of NRP1

**Figure 3:**
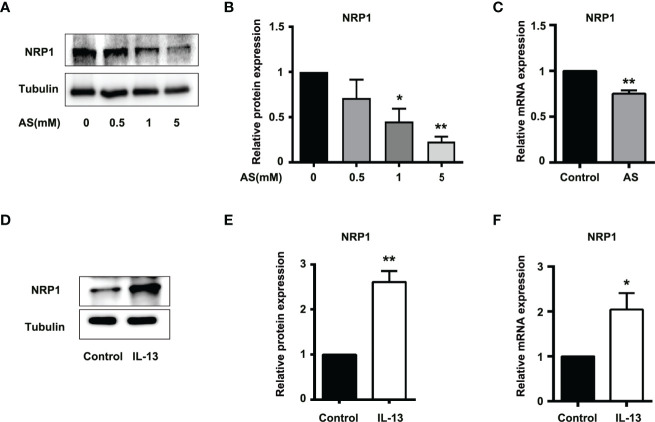
STAT6-mediated NRP1 expression in HUVECs. **(A)** Expression of NRP1 was detected by western blotting in HUVECs treated with 0.5, 1, or 5 μM AS for 24 (h)** (B)** Relative NRP1 expression normalized with tubulin after AS treatment, n=3, *P<0.05 vs. control, **P<0.01 vs. control. **(C)** Expression of *NRP1* was detected by qPCR in HUVECs treated with AS (1 μM) for 24 h, n=3, **P<0.01 vs. control. **(D)** Expression of NRP1 was detected by western blotting in HUVECs treated with IL-13 (50 ng/mL) for 24 (h)** (E)** Relative NRP1 expression normalized with tubulin after IL-13 treatment, n=3, *P<0.05 vs. control, **P<0.01 vs. control. **(F)** Expression of NRP1 was detected by qPCR in HUVECs treated with IL-13 (50 ng/mL) for 24 h, n=3, **P<0.01 vs. control.

**Figure 4:**
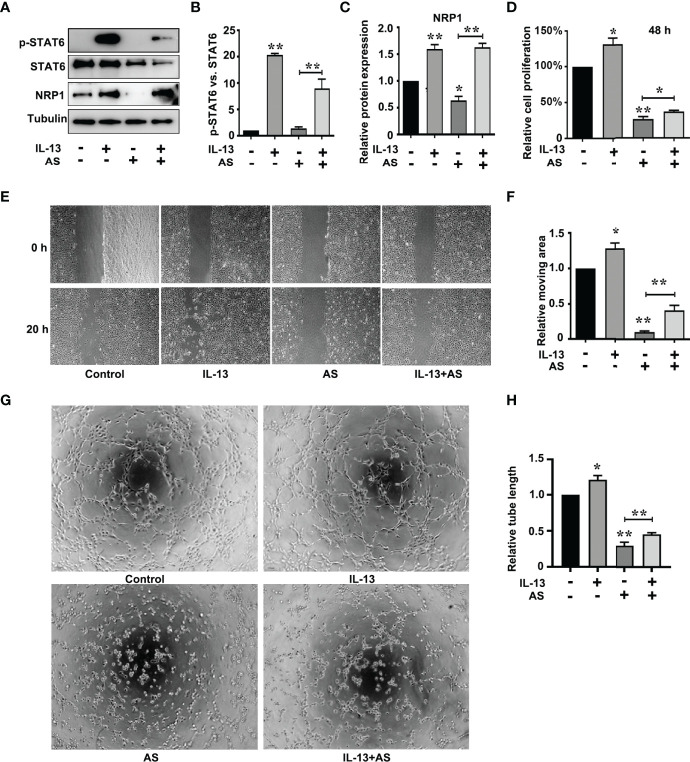
STAT6-mediated NRP1 expression and promotes migration, proliferation, and tube-formation in HUVECs. **(A)** Phosphorylation of STAT6, STAT6, and NRP1 were detected by western blotting in HUVECs treated with AS (1 μM), IL-13 (50 ng/mL), and AS combined with IL-13 for 24 (h)** (B)** Relative STAT6 phosphorylation level was normalized with STAT6, n=3, **P<0.01 vs. control. **(C)** Relative NRP1 expression normalized with tubulin after AS and IL-13 treatment, n=3, *P<0.05 vs. control, **P<0.01 vs. control. **(D)** The relative cell proliferation was detected by MTT in HUVECs treated with AS (1 μM), IL-13 (50 ng/mL), and AS combined with IL-13 for 48 h, n=5, *P<0.05 vs. control, **P<0.01 vs. control. **(E, F)** The typical images and the relative moving area of HUVECs treated with AS (1 μM), IL-13 (50 ng/mL), and AS combined with IL-13 for 20 h, n=5, *P<0.05 vs. control, **P<0.01 vs. control. **(G, H)** Typical images and relative tube length of HUVECs treated with AS (1 μM), IL-13 (50 ng/mL), and AS combined with IL-13 for 24 h, n=5, *P<0.05 vs. control, **P<0.01 vs. control.

The correlation scores between the mRNA levels of *STAT6* and *NRP1* was the highest in these candidate genes. To further determine whether NRP1 expression was regulated by STAT6, we detected NRP1 expression in the presence of STAT6 inhibitor (AS) or activator (IL-13). We found that AS treatment significantly reduced the protein and mRNA expression of NRP1 (**Figures 3A–C**). In contrast, IL-13 increased NRP1 protein and mRNA expression (**Figures 3D–F**). In addition, NRP1 expression was detected by qPCR and western blotting when HUVECs were treated with AS (1 μM), IL-13 (50 ng/mL), and AS combined with IL-13. AS reduced the phosphorylation of STAT6, which was induced by IL-13 (**Figures 4A, B**). Furthermore, the increasing NRP1 was inhibited by AS treatment (**Figure 4C**). The mRNA level of NRP1 was reduced by AS treatment, and increased by IL-13 treatment. AS reduced NRP1 expression even in the presence of IL-13 (**Figure S3**). Knockdown of STAT6 by STAT6 siRNA was used to evaluate the effect of IL-13 on NRP1 expression. Western blotting showed that knockdown of STAT6 reduced NRP1 expression even in the presence of IL-13 (**Figure S4**). Taken together, these findings suggest that the STAT6 signal pathway promotes NRP1 expression.

### STAT6/NRP1 Signal Pathway Regulates EC Proliferation, Migration, and Tube-Formation

To explore the role of the STAT6 signaling pathway in EC angiogenesis, we assessed the effect of AS (1 μM), IL-13 (50 ng/mL), or AS combined with IL-13 treatment on EC proliferation, migration, and tube-formation. IL-13 treatment for 24 h did not affect the proliferation of HUVECs. When the proliferation time was prolonged to 48 h, IL-13 treatment modestly increased the proliferation of HUVECs (**Figure 4D**). For the AS and IL-13 co-treatment group, AS treatment abolished the promoting effect of IL-13 on HUVEC proliferation (**Figure 4D**). Moreover, migration of HUVEC was also modestly increased by IL-13 treatment, and AS treatment abolished the promoting effect of IL-13 on HUVEC migration (**Figures 4E, F**). Tube-formation was increased by IL-13 treatment, and AS treatment abolished the promoting effect of IL-13 on HUVEC tube-formation (**Figures 4G, H**). Similar results were obtained by disrupting STAT6 expression. Knockdown of STAT6 also inhibited EC proliferation. Furthermore, knockdown of STAT6 reduced migration, and tube-formation, induced by IL-13 (**Figure S1**). In addition, knockdown of NRP1 by siRNA also reduced EC proliferation, and inhibited EC migration and tube-formation even in the presence of IL-13 (**Figures S5** and **S6**). These results suggested that the STAT6 signaling pathway promotes EC angiogenesis.

### STAT6 Regulates NRP1 Expression by Binding to its Promoter

**Figure 5:**
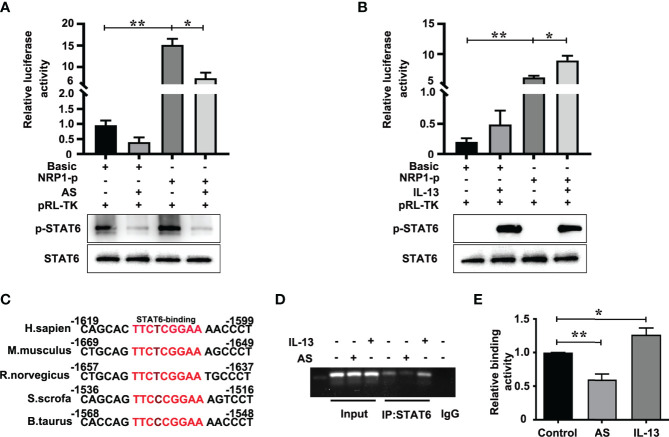
STAT6 directly targets the *NRP1* promoter in HUVECs. **(A)** Luciferase assay in HUVECs after transfection with pGL3-Basic (Basic) or pGL3-*NRP1* prompter (*NRP1*-p) and then treated with or without AS (1 μM AS) for 24 (h) The level of phosphorylation of STAT6 and total STAT6 were also detected by western blotting, n=3, *P<0.05, *NRP1*-p vs. *NRP1*-p treated with AS, **P<0.01 *NRP1*-p vs. basic. **(B)** Luciferase assay in HUVECs after transfection with pGL3-Basic (Basic) or pGL3-*NRP1* prompter (*NRP1*-p) and then treated with or without IL-13 (50 ng/mL) for 24 (h) The level of phosphorylation of STAT6 and total STAT6 were also detected by western blotting, n=3, *P<0.05, *NRP1*-p vs. *NRP1*-p treated with IL-13, **P<0.01 *NRP1*-p vs. basic. **(C)** The conserved sequence of STAT6 binding sites in the *NRP1* promoter sequence were compared between different species. **(D, E)** ChIP assay was performed using anti-STAT6 antibody in HUVECs after treatment with AS (1 μM) or IL-13 (50 ng/mL) for 24 h, and then amplified by PCR, n=3, *P<0.05 vs. control, **P<0.01 vs. control.

The positive role of NRP1 in angiogenesis has been wide verified (25, 26). To discover the mechanism of STAT6-regulated NRP1 expression, we first analyzed the effect of AS and IL-13 treatment on the promoter activity of *NRP1*. Luciferase assay showed that the promoter activity of *NRP1* is much higher than the promoter activity of pGL3-basic, and AS treatment significantly inhibited the promoter activity of *NRP1* when compared with non-AS treatment (**Figure 5A**). IL-13 increased the phosphorylation of STAT6, and the promoter activity of *NRP1* was enhanced by IL-13 incubation (**Figure 5B**). Compared with the control group, co-treatment with both IL-13 and AS reduced the promoter activity of *NRP1*, but there was no significant difference between the co-treatment group and AS treatment group (**Figure S7**). Taken together, these results showed that inhibition of STAT6 reduced promoter activity of *NRP1.* We further searched the binding sequences of STAT6 in the promoter region of human *NRP1* and assessed the homologous sequences of STAT6 binding sites between different species. Sequence analysis indicated that one presumptive STAT6 binding site located at the promoter regions of human *NRP1* was from -1613 to -1605. These sequences were highly homologous with *M. musculus* (-1663 to -1655), *R. norvegicus* (-1651 to -1643), *S. scrofa* (-1530 to -1522), and *B. taurus* (-1562 to -1554) (**Figure 5C**). Therefore, a ChIP primer was designed according to the regions from -1613 to -1605 of the human *NRP1* promoter. We found that enrichment of STAT6 at the *NRP1* promoter; AS treatment reduced this enrichment, and incubation with IL-13 modestly enhanced the enrichment (**Figures 5D, E**). These results suggest that STAT6 binds to the promoter region of *NRP1* and enhances NRP1 expression.

### Inhibition of STAT6 Activity Suppresses Tumor Angiogenesis and NRP1 Expression *In Vivo*

**Figure 6:**
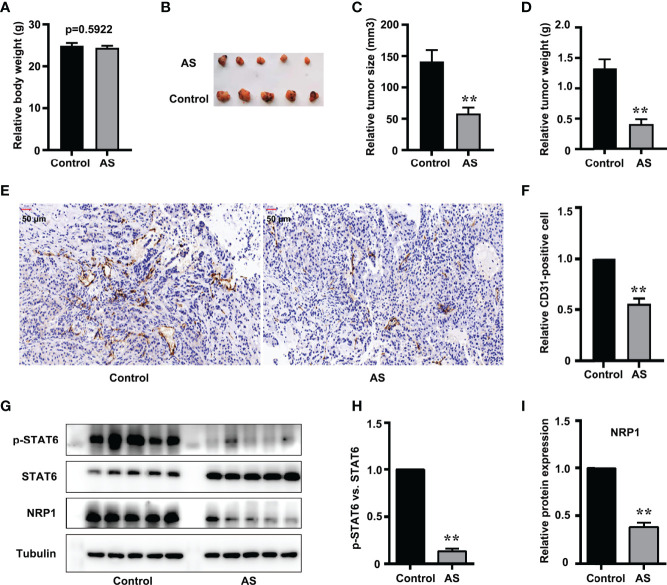
Inhibition of STAT6 reduces xenograft growth and tumor angiogenesis *in vivo*. **(A)** Body weights were measured before all nude mice were killed. **(B)** Resected subcutaneous tumors from indicated A549 cell-injected groups in nude mice. **(C, D)** Tumor weight and tumor volume were measured, n=5, **P<0.01 vs. control. **(E, F)** Vascular intensity by A549 cell-derived tumor treated with AS (25 mg/kg body weight) was evaluated by IHC with anti-CD31 antibody. Scale bar: 50 μm. n=5, **P<0.01 vs. control. **(G)** Phosphorylation of STAT6, STAT6, and NRP1 were detected by western blotting in tumor tissue. **(H)** Relative STAT6 phosphorylation level normalized with STAT6, n=5, **P<0.01 vs. control. **(I)** Relative NRP1 expression normalized with tubulin, n=5, **P<0.01 vs. control.

Nude mice xenograft assay was performed to further evaluate the *in vivo* effect of STAT6 on angiogenesis. After being administered with AS for 30 days, we found that the bodyweight of the xenograft mice was not affected by AS treatment (**Figure 6A**), and the tumor size and tumor weight were suppressed by AS treatment (**Figures 6B–D**). These results confirmed that AS treatment inhibited the growth of tumor xenografts in nude mice. We further explored the effects of AS treatment on tumor angiogenesis. CD31 was used as an EC marker, and IHC assay found that AS administration reduced vascular density (**Figures 6E, F**). Moreover, the expression of STAT6 and NRP1 were also detected in subcutaneous tumors. As expected, the phosphorylation of STAT6 was down-regulated by AS administration, and the expression of NRP1 was also reduced (**Figures 6G–I**). *In situ* analysis of NRP1 level by immunostaining and immunofluorescence staining showed that AS treatment reduced the expression of NRP1 was in the entire cell type compared with control group (**Figure S8**). These results confirmed that STAT6 regulates the expression of NRP1 and affects tumor angiogenesis *in vivo.*

## Discussion

**Figure 7:**
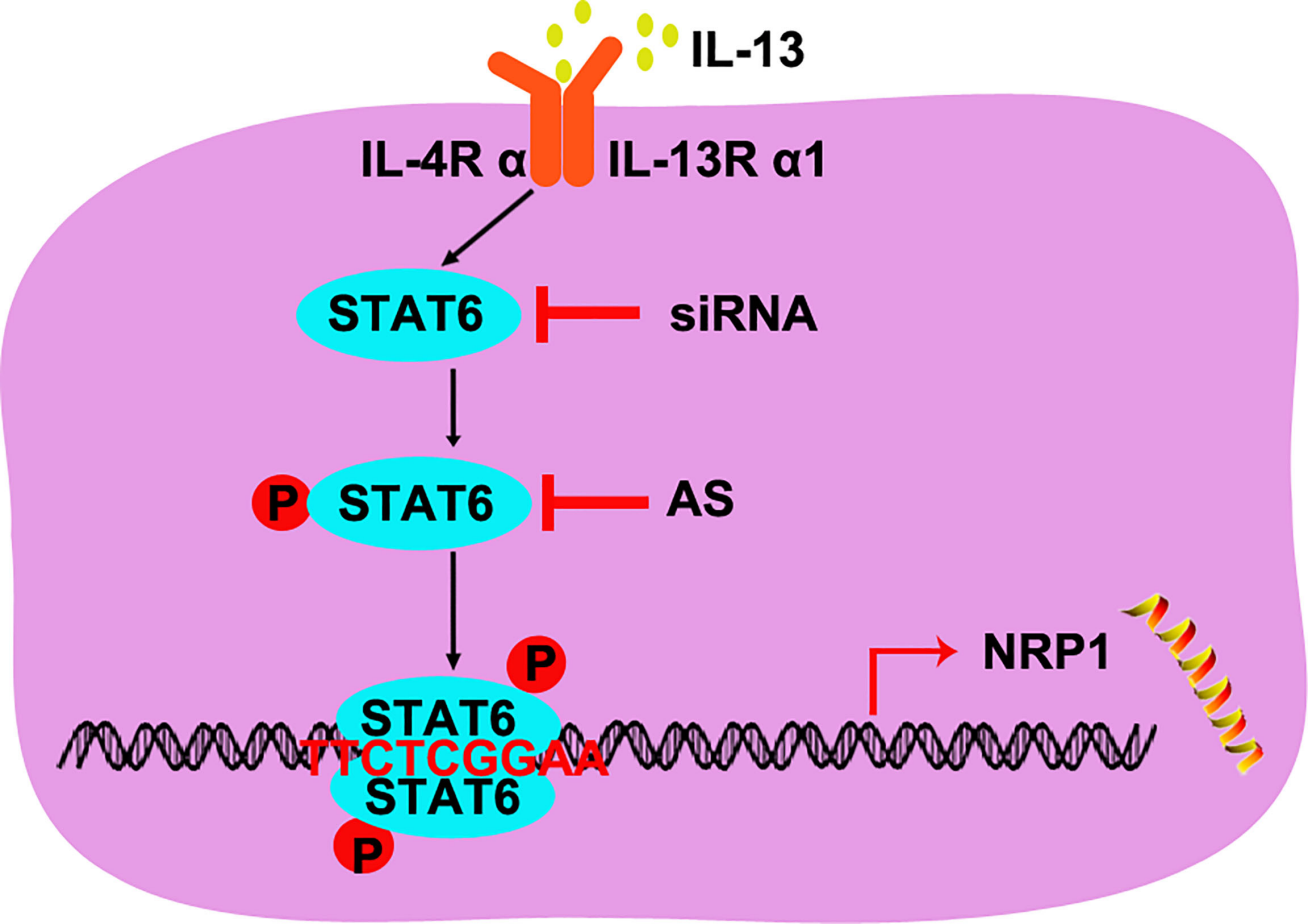
A schematic underlying the mechanism of STAT6-promoted NRP1 expression in HUVECs.

In 1971, Folkman reported that solid neoplasms are always accompanied by angiogenesis, and the new capillary supported tumor growth and metastasis (27). Anti-angiogenesis was then developed as a strategy for tumor treatment, and it has already been used in anti-tumor combined therapy. Apatinib, an inhibitor of VEGFR, and bevacizumab, humanized anti-VEGF monoclonal antibodies, have been used to treat non-small-cell lung cancer combined with PD-L1 antibody or chemotherapy (28). More anti-angiogenesis targets are needed for the cocktail of antibodies or inhibitors. In our study, we found that STAT6 was a potential target for anti-angiogenesis therapy. Activation of STAT6 promoted proliferation, migration, and tube-formation of HUVECs and inhibition of STAT6 reduced proliferation, migration, and tube-formation of HUVECs. We also confirmed the mechanisms of STAT6 affecting EC function by binding to the promoter of *NRP1* and increasing NRP1 expression (**Figure 7**). Thus, STAT6 is a potential therapeutic target for anti-tumor angiogenesis.

STAT6 is generally recognized as a transcription factor that promotes M2 polarization of macrophages (29). Recently, the effect of STAT6 on angiogenesis has also been reported. VEGF treatment increases the nuclear translocation of STAT6 and promotes EC migration (30). Silencing STAT6 with siRNA inhibits VEGF-induced *in vivo* angiogenesis (30). In human LUAC squamous cell carcinoma, high expression of STAT6 has been detected (31). STAT6 deficiency with siRNA inhibits carcinogen-induced lung cancer growth and improves prognosis in cancer transplantation mice model (31). Inhibitor of STAT6 (AS1517499) combined with 5-fluorouracil markedly reduce the tumor load (32). In an orthotopic 4T1 mammary carcinoma mouse model, AS1517499 treatment attenuated tumor growth and early liver metastasis (33). In our study, AS1517499 administration for both HUVECs and nude mouse xenograft showed that inhibition of STAT6 reduced proliferation, migration, and tube-formation of HUVECs and reduced tumor size and tumor angiogenesis in a mouse xenograft model. Our results are similar to previous reports.

IL-13 is an inflammatory factor that has multiple functions including regulation of tumor development (11). IL-13 activates STAT6 and promotes the M2 polarization of macrophages. The conditioned medium of IL-13-treated M2 macrophages induces tumor invasion, migration, and angiogenesis of A549 and H1299 cells (34). In our study, IL-13 was also used to incubate HUVECs, and we found that IL-13 treatment modestly promoted the proliferation and migration of HUVECs. Co-treatment with AS1517499 blocked the promotion of proliferation and migration induced by IL-13. These data suggested the target role of STAT6 for anti-angiogenesis therapy.

To explore the mechanisms of STAT6-mediated angiogenesis, we further detected the expression of pro-angiogenic genes. Inhibition of STAT6 activity reduced the expression of *CCL2*, *MMP2*, *KDR*, *NRP1*, *VEGFA*, *SPHK1*, *SMAD5*, *EDN1*, and *PTGS1*. Some of these genes are reportedly regulated by STAT6. For instance, activation of STAT6 binds upstream of the VEGF promoter (from -338 to -305 bp) in mouse ECs and promotes VEGF expression (30). IL-13 selectively increases CCL2 expression and secretion through IL-4Rα and STAT6 phosphorylation in HUVECs (35). Inhibition of IL-4Rα/STAT6 signal pathway by anti IL-4Rα antibody reduces CCL2 expression (35). IL-13 also promotes EC angiogenesis by activating STAT6 and then increasing the expression of vascular cell adhesion molecule-1 (VCAM-1) and soluble VCAM-1 (36). In HT-1080 tumor cells, progesterone-induced blocking factor (PIBF) treatment increases STAT6 phosphorylation, and inhibition of PIBF with siRNA significantly reduces MMP2 expression (37). These data suggest that STAT6 regulates lung tumor angiogenesis by mediating the expression of pro-angiogenic genes.

NRP1 is identified as a receptor for VEGFA165 and class-3 semaphorins, and is crucial for mouse and zebrafish vascular development as well as pathological angiogenesis (25, 26). Although semaphorin-NRP1 signaling is not essential for vascular development in mouse embryos, semaphorin 3A (SEMA3A) participates in modulating tumor angiogenesis in mouse cancer models (38). It has been reported that SEMA 3A inhibited VEGF-mediated angiogenesis in an NRP1-dependent manner (39), and over-expression of SEMA 3A induced EC apoptosis and promoted vascular maturation by recruiting pericytes and monocytes expressing NRP1 (40, 41). Both SEMA 3A and VEGF increased vascular permeability in an NRP1-dependent manner; however, they used distinct downstream effectors (39). Another study also reported that SEMA3A could induce permeability signaling by NRP2 and VEGFR1, independent of NRP1 (42). Therefore, NRP1 is essential for transmitting both VEGF and SEMA 3A signals to regulate tumor angiogenesis. It has been reported that recombinant rat NRP-1 chimera treatment increases tubular morphogenesis and cell migration of human dermal microvascular endothelial cells (HDMECs) and HUVECs (43). In patients with non-small cell lung cancer (NSCLC), high expression of NRP1 has shorter overall survival than in patients with low expression of NRP1. Inhibition of NRP1 suppresses tumor migration and angiogenesis (44). In our study, the correlation score between the mRNA levels of *STAT6* and *NRP1* was the highest in the potential target molecules. We found that IL-13 treatment up-regulated the expression of NRP1, and AS1517499 administration reduced the expression of NRP1 in HUVECs. Interestingly, AS1517499 blocked the promotion of NRP1 expression induced by IL-13. All this evidence indicates that NRP1 is a new target for STAT6 in regulating EC angiogenesis.

We extensively explored the underlying mechanisms by which STAT6 regulates NRP1 expression. As an important component of the VEGF signal pathway, multiple regulatory elements regulate NRP1 expression in a cooperative manner. There are one AP1 element and two SP1 elements that contributed to constitutive and tumor promoter-induced promoter activity of *NRP1* in HeLa cells (45). TEA domain transcription factor (TEAD) binding motif is also present in the promoter region of *NRP1*, and the expression of NRP1 is regulated by TEAD in hepatocellular carcinoma (HCC) (46). In the present study, we found the binding site of STAT6 (TTCnnnGGA sequence) (12) in the promoter region of *NRP1*, which was conserved in many species. Luciferase assay demonstrated that activation of STAT6 by IL-13 increased the transcription activity of *NRP1* promoter, while inhibition of STAT6 activity by AS1517499 significantly reduced *NRP1* promoter transcription activity. ChIP assay demonstrated that STAT6 is directly bound to promoter region of *NRP1* in HUVECs.

The impact of STAT6 inhibition on NRP1 mRNA levels (**Figure 3C**) is modest. The involvement of other factors involved in the regulation of NRP1 expression cannot be overlooked. In fact, both SP1 and HIF-1α positively regulate NRP1 expression in tumor cells (47, 48). STAT6 interacted with SP1 and increased the expression of p21 and p27 in promoting breast cancer cell proliferation (49). In B-lymphoblastoid cell line, type I IFN-activated STAT6 could increase Sp1 and BCL6 through STAT2 and exert the anti-proliferative effects (50). In glioma cells, STAT6 negatively regulated HIF-1α expression *via* mTOR/S6K/S6 axis (51). Although the binding of STAT6 to the NRP1 promoter was detected in our study, the experimental results indicated that other mediators participated in the regulation of NRP1 expression by STAT6. This regulation could also rely on an indirect mechanism. As discussed earlier, STAT6 can influence tumor angiogenesis through factors such as VCAM-1 and MMP (36, 37), and NRP1 is not the only target molecule of STAT6.

Taken together, our results indicate that STAT6 promotes EC proliferation, migration, and tube-formation. In addition, STAT6 upregulates NRP1 expression in ECs and promotes angiogenesis. Therefore, STAT6 may be considered potential therapeutic target for anti-angiogenic therapy.

## Data Availability Statement

The raw data supporting the conclusions of this article will be made available by the authors, without undue reservation.

## Ethics Statement

The animal study was reviewed and approved by Animal Care and Use Committee of the First Affiliated Hospital of Shandong First Medical University.

## Author Contributions

PG and JL conceived and designed the experiments. PG and GR performed the experiments. PG, GR, and JJL analyzed the data. PG, GR, and JL wrote the paper. JL reviewed and edited the paper. All authors contributed to the article and approved the submitted version.

## Funding

This study was supported by the Natural Science Foundation of Shandong Province (ZR2019BH007), the foundation of Qianfoshan Hospital (QYPY2020NSFC0820), the Jinan City’s Science and Technology Innovation Program of Clinical Medicine (202019175), the National Nature Science Foundation of China (81873473), Academic Promotion Program of Shandong First Medical University (2019QL014), and Shandong Taishan Scholarship (JL).

## Conflict of Interest

The authors declare that the research was conducted in the absence of any commercial or financial relationships that could be construed as a potential conflict of interest.

## Publisher’s Note

All claims expressed in this article are solely those of the authors and do not necessarily represent those of their affiliated organizations, or those of the publisher, the editors and the reviewers. Any product that may be evaluated in this article, or claim that may be made by its manufacturer, is not guaranteed or endorsed by the publisher.

## Supplementary Material

Supplementary Figure 1 Knockdown of STAT6 inhibits HUVEC proliferation, tube-formation, and migration. **(A)** The expression of STAT6 was detected by western blotting in HUVECs after transfected with STAT6 siRNA for 48 h. **(B)** The relative STAT6 expression normalized with tubulin, n=3, **P*<0.05 vs. control. **(C)** The mRNA level of STAT6 was detected by qPCR in HUVECs after transfected with STAT6 siRNA for 48 h, n=3, ***P*<0.01 vs. control. **(D)** After transfected with STAT6 siRNA for 48 h, HUVECs were cultured for 0 h, 24 h, 48 h, and 72 h, the cell growth curve of HUVECs were detected by MTT, n=5, ***P*<0.01 vs. control. **(E, F)** After transfected with STAT6 siRNA for 48 h, The typical images and relative tube length of HUVECs were detected in the presence or absence of IL-13 (50 ng/mL), n=5, **P*<0.05 vs. control, NS, no significant difference. **(G, H)** After transfected with STAT6 siRNA for 48 h, the typical images and the relative moving area of HUVECs treated with or without IL-13 (50 ng/mL) for 20 h, n=5, ***P*<0.01 vs. control, NS denotes no significant difference.

Supplementary Figure 2 STAT6 expression in LUAD tissues is positively correlated with *MMP2*, *KDR*, *NRP1*, *VEGFA*, *CCL2*, *SPHK1*, *SMAD5*, *EDN1*, and *PTGS1* levels. A-I, Scatter plot analysis of the correlation between mRNA levels of *STAT6* and *MMP2*, *KDR*, *NRP1*, *VEGFA*, *CCL2*, *SPHK1*, *SMAD5*, *EDN1* or *PTGS1* in LUAD of tissues.

Supplementary Figure 3 AS treatment reduces NRP1 mRNA levels in presence of IL13 stimulation. The mRNA levels of NRP1 were detected by qPCR in HUVECs treated with AS (1 μM), IL-13 (50 ng/mL), and AS combined with IL-13 for 24 h. Relative NRP1 mRNA levels were normalized with actin, n=3, ***P*<0.01 vs. control, NS denotes no significant difference.

Supplementary Figure 4 Knockdown of STAT6 reduces NRP1 expression in the presence of IL-13 in HUVECs. **(A)** Phosphorylation of STAT6, STAT6, and NRP1 were detected by western blotting in STAT6 knockdown of HUVECs treated with or without IL-13 (50 ng/mL). **(B)** Relative NRP1 expression was normalized with Tubulin, n=3, **P*<0.05 vs. control, NS denotes no significant difference.

Supplementary Figure 5 Knockdown of NRP1 by NRP1 siRNA 2 inhibits HUVEC migration, proliferation, and tube-formation. **(A)** The expression of NRP1 was detected by western blotting in HUVECs after transfected with NRP1 siRNA 2 for 48 h. **(B)** The relative NRP1 expression normalized with tubulin, n=3, **P*<0.05 vs. control. **(C)** The mRNA level of NRP1 was detected by qPCR in HUVECs after transfected with NRP1 siRNA 2 for 48 h, n=3, ***P*<0.01 vs. control. **(D)**, After transfected with NRP1 siRNA 2 for 48 h, HUVECs were cultured for 0 h, 24 h, 48 h, and 72 h, the cell growth curve of HUVECs were detected by MTT, n=5, ***P*<0.01 vs. control. **(E, F)** After transfected with NRP1 siRNA 2 for 48 h, The typical images and relative tube length of HUVECs were detected in the presence or absence of IL-13 (50 ng/mL) n=5, ***P*<0.01 vs. control, NS denotes no significant difference. **(G, H)** After transfected with NRP1 siRNA 2 for 48 h, the typical images and the relative moving area of HUVECs treated with or without IL-13 (50 ng/mL) for 20 h, n=5, **P*<0.05 vs. control, NS denotes no significant difference.

Supplementary Figure 6 Knockdown of NRP1 by NRP1 siRNA 3 inhibits HUVEC migration, proliferation, and tube-formation. **(A)** The expression of NRP1 was detected by western blotting in HUVECs after transfected with NRP1 siRNA 3 for 48 h. **(B)** The relative NRP1 expression normalized with tubulin, n=3, **P*<0.05 vs. control. **(C)** The mRNA level of NRP1 was detected by qPCR in HUVECs after transfected with NRP1 siRNA 3 for 48 h, n=3, ***P*<0.01 vs. control. **(D)**, After transfected with NRP1 siRNA 3 for 48 h, HUVECs were cultured for 0 h, 24 h, 48 h, and 72 h, the cell growth curve of HUVECs were detected by MTT, n=5, ***P*<0.01 vs. control. **(E, F)** After transfected with NRP1 siRNA 3 for 48 h, The typical images and relative tube length of HUVECs were detected in the presence or absence of IL-13 (50 ng/mL) n=5, ***P*<0.01 vs. control, NS denotes no significant difference. **(G, H)** After transfected with NRP1 siRNA 3 for 48 h, the typical images and the relative moving area of HUVECs treated with or without IL-13 (50 ng/mL) for 20 h, n=5, **P*<0.05 vs. control, NS denotes no significant difference.

Supplementary Figure 7 Inhibition of STAT6 reduces the promoter activity of NRP1 in the presence of IL-13. Luciferase assay in HUVECs after transfection with pGL3-*NRP1* prompter (*NRP1*-p) and then treated with AS (1 μM), IL-13 (50 ng/mL), and AS combined with IL-13 for 24 h, n=5, ***P*<0.01 vs. control, NS denotes no significant difference.

Supplementary Figure 8 Inhibition of STAT6 reduces expression of NRP1 *in vivo*. **(A)** The expression of NRP1 in A549 cell-derived tumor treated with or without AS (25 mg/kg body weight) was evaluated by IHC with anti-NRP1 antibody. Scale bar: 50 μm. **(B)** Relative NRP1 positive cell, n=5, ***P*<0.01 vs. control. **(C)** The expression of NRP1 in A549 cell-derived tumor treated with or without AS (25 mg/kg body weight) was evaluated by immunofluorescence with anti-NRP1 antibody (green) and anti-CD31 antibody (red). DAPI was used to display the nucleus. Scale bar: 50 μm.

## Abbreviations

EDN1, endothelin-1; KDR, kinase insert domain-containing receptor; MCP-1/CCL2, monocyte chemoattractant protein-1; MMP2, matrix metalloproteinase-2; NRP1, neuropilin-1; PTGS1/COX1, cyclooxygenase 1; SMAD5, mothers against DPP homolog 5; SPHK1, sphingosine kinase 1; VEGFA, vascular endothelial growth factor A.
