# Supplementary figures and images for: STAT6 Upregulates NRP1 Expression in Endothelial Cells and Promotes Angiogenesis
Source: Front Oncol. 2022 May 5;12:823377. doi: 10.3389/fonc.2022.823377 (PMC9117725; doi:10.3389/fonc.2022.823377)

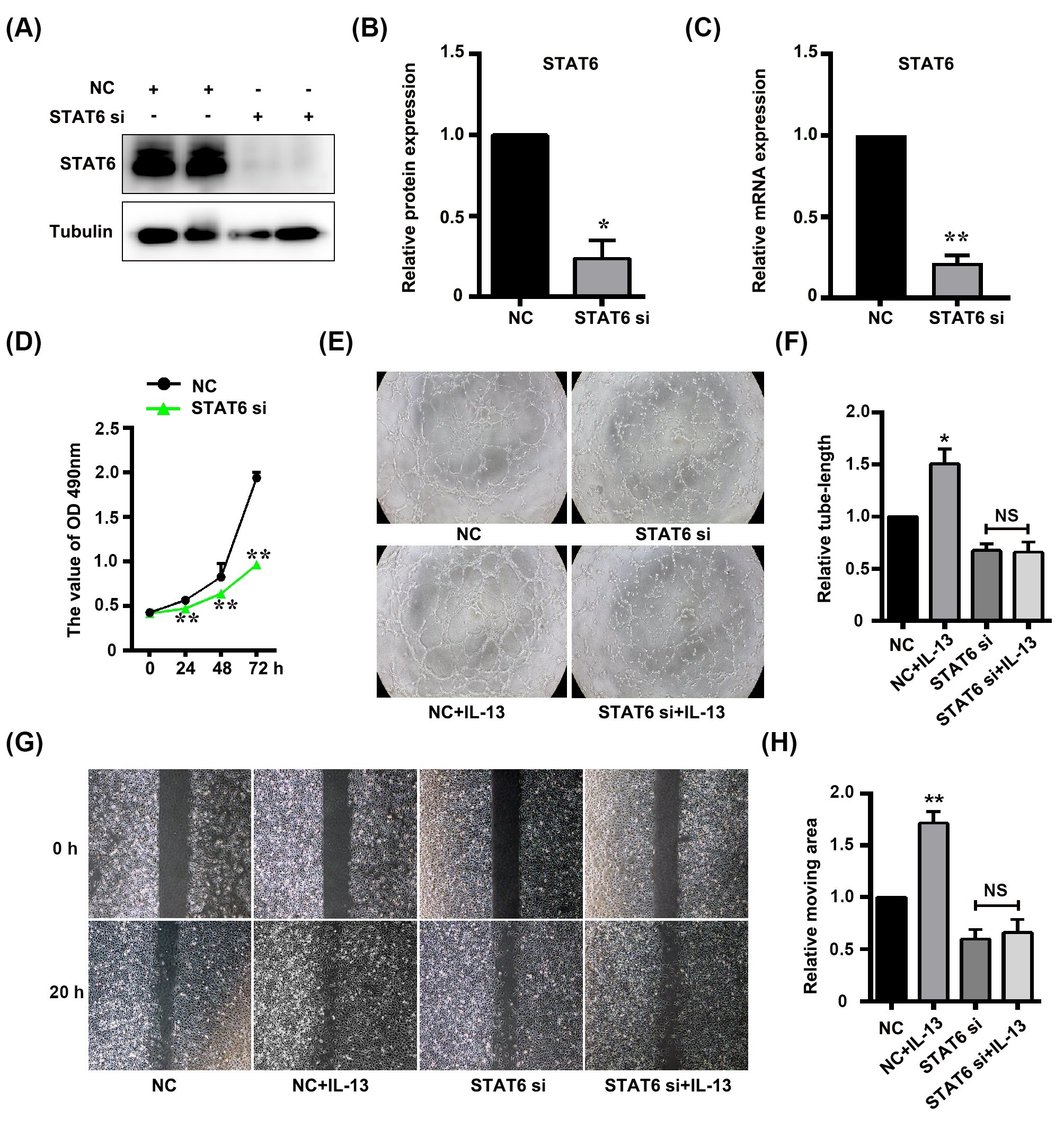

Supplement: Supplementary Figure 1 — Knockdown of STAT6 inhibits HUVEC proliferation, tube-formation, and migration. (A) The expression of STAT6 was detected by western blotting in HUVECs after transfected with STAT6 siRNA for 48 h. (B) The relative STAT6 expression normalized with tubulin, n=3, *P<0.05 vs. control. (C) The mRNA level of STAT6 was detected by qPCR in HUVECs after transfected with STAT6 siRNA for 48 h, n=3, **P<0.01 vs. control. (D) After transfected with STAT6 siRNA for 48 h, HUVECs were cultured for 0 h, 24 h, 48 h, and 72 h, the cell growth curve of HUVECs were detected by MTT, n=5, **P<0.01 vs. control. (E, F) After transfected with STAT6 siRNA for 48 h, The typical images and relative tube length of HUVECs were detected in the presence or absence of IL-13 (50 ng/mL), n=5, *P<0.05 vs. control, NS, no significant difference. (G, H) After transfected with STAT6 siRNA for 48 h, the typical images and the relative moving area of HUVECs treated with or without IL-13 (50 ng/mL) for 20 h, n=5, **P<0.01 vs. control, NS denotes no significant difference. [file Image_1.tif]

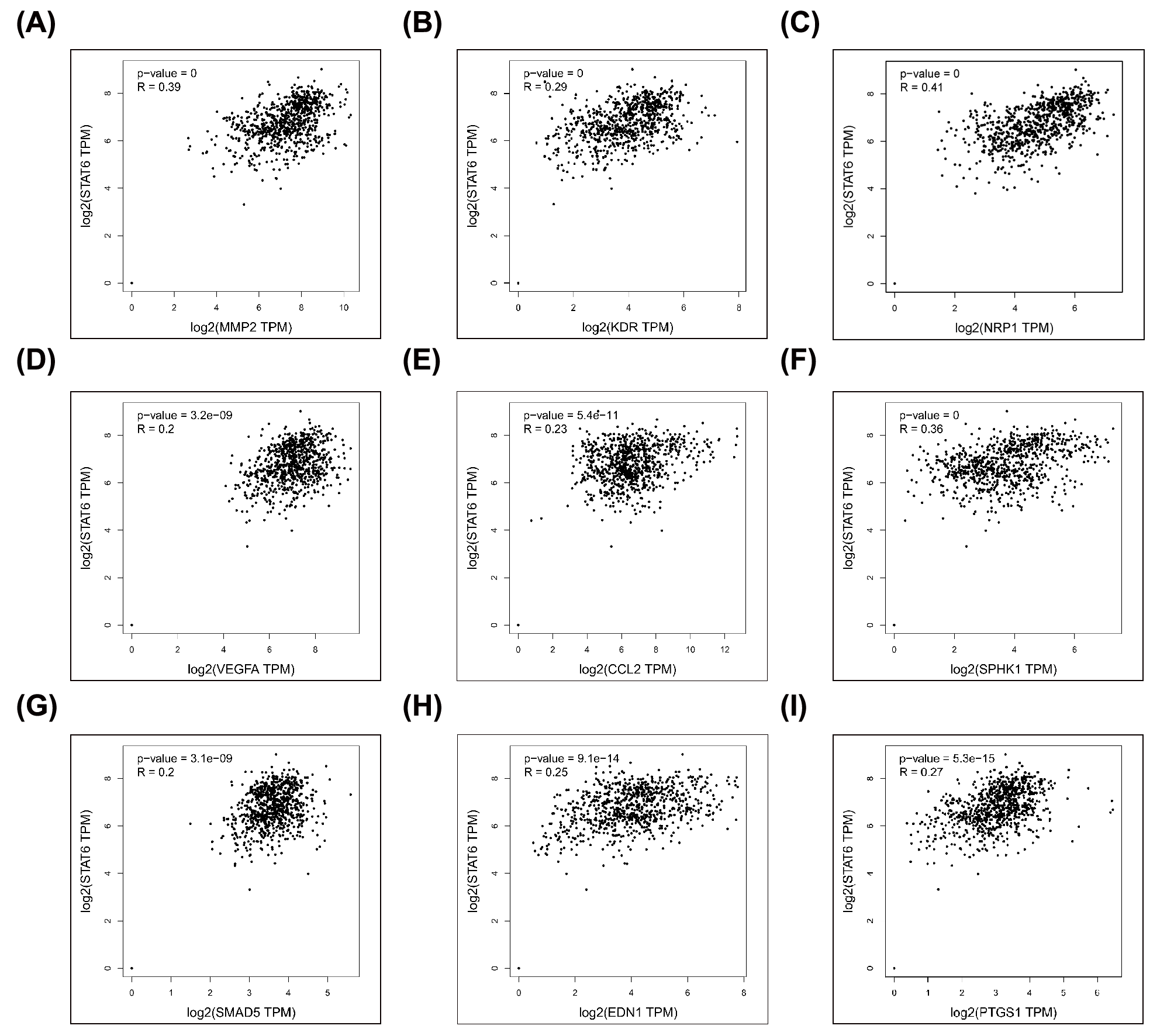

Supplement: Supplementary Figure 2 — STAT6 expression in LUAD tissues is positively correlated with MMP2, KDR, NRP1, VEGFA, CCL2, SPHK1, SMAD5, EDN1, and PTGS1 levels. A-I, Scatter plot analysis of the correlation between mRNA levels of STAT6 and MMP2, KDR, NRP1, VEGFA, CCL2, SPHK1, SMAD5, EDN1 or PTGS1 in LUAD of tissues. [file Image_2.tif]

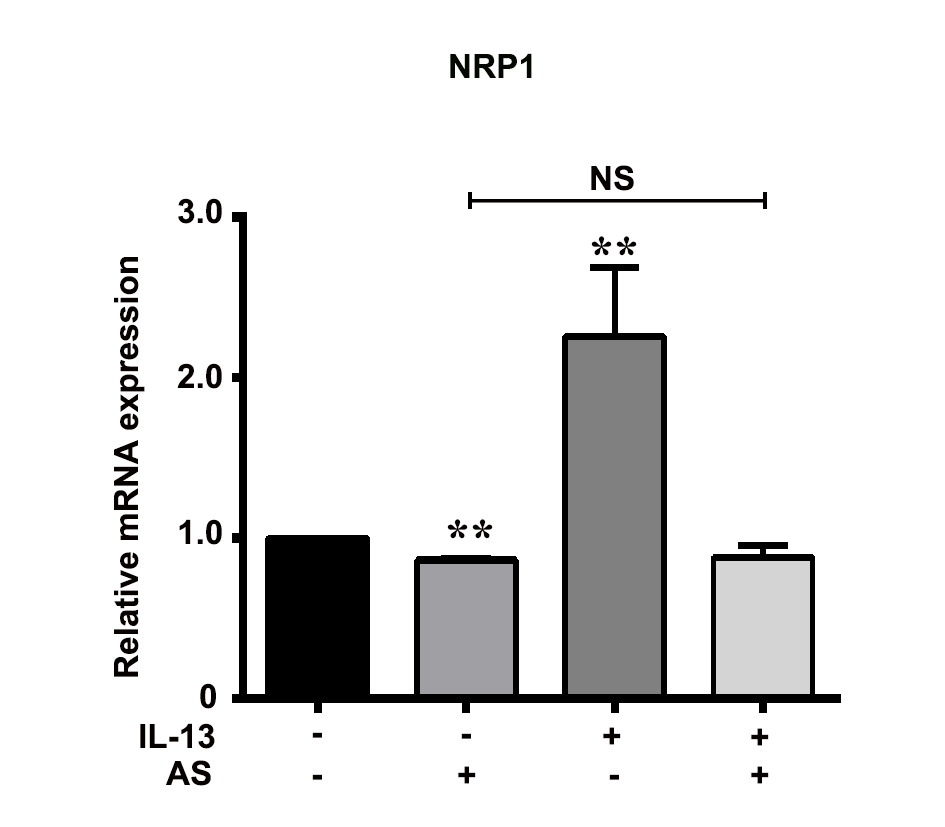

Supplement: Supplementary Figure 3 — AS treatment reduces NRP1 mRNA levels in presence of IL13 stimulation. The mRNA levels of NRP1 were detected by qPCR in HUVECs treated with AS (1 μM), IL-13 (50 ng/mL), and AS combined with IL-13 for 24 h. Relative NRP1 mRNA levels were normalized with actin, n=3, **P<0.01 vs. control, NS denotes no significant difference. [file Image_3.tif]

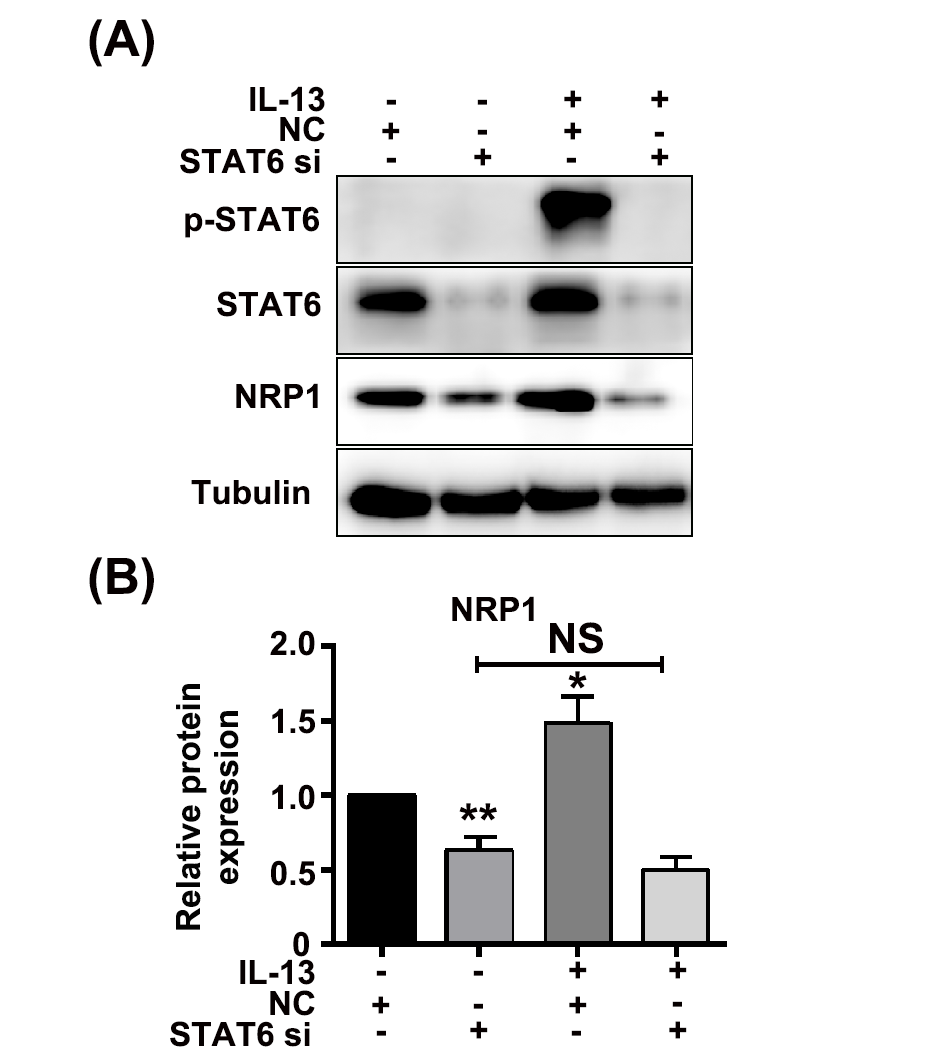

Supplement: Supplementary Figure 4 — Knockdown of STAT6 reduces NRP1 expression in the presence of IL-13 in HUVECs. (A) Phosphorylation of STAT6, STAT6, and NRP1 were detected by western blotting in STAT6 knockdown of HUVECs treated with or without IL-13 (50 ng/mL). (B) Relative NRP1 expression was normalized with Tubulin, n=3, *P<0.05 vs. control, NS denotes no significant difference. [file Image_4.tif]

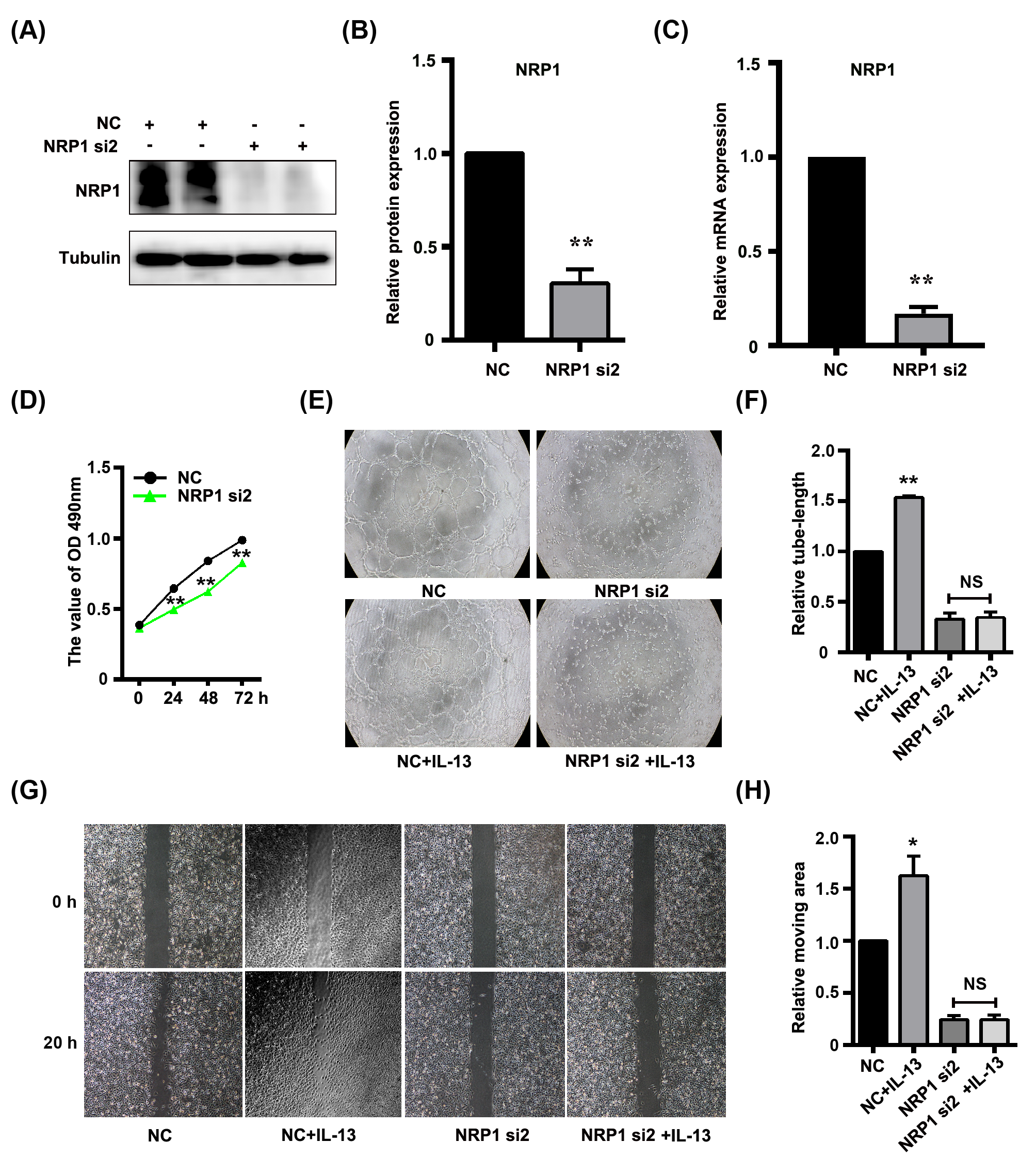

Supplement: Supplementary Figure 5 — Knockdown of NRP1 by NRP1 siRNA 2 inhibits HUVEC migration, proliferation, and tube-formation. (A) The expression of NRP1 was detected by western blotting in HUVECs after transfected with NRP1 siRNA 2 for 48 h. (B) The relative NRP1 expression normalized with tubulin, n=3, *P<0.05 vs. control. (C) The mRNA level of NRP1 was detected by qPCR in HUVECs after transfected with NRP1 siRNA 2 for 48 h, n=3, **P<0.01 vs. control. (D), After transfected with NRP1 siRNA 2 for 48 h, HUVECs were cultured for 0 h, 24 h, 48 h, and 72 h, the cell growth curve of HUVECs were detected by MTT, n=5, **P<0.01 vs. control. (E, F) After transfected with NRP1 siRNA 2 for 48 h, The typical images and relative tube length of HUVECs were detected in the presence or absence of IL-13 (50 ng/mL) n=5, **P<0.01 vs. control, NS denotes no significant difference. (G, H) After transfected with NRP1 siRNA 2 for 48 h, the typical images and the relative moving area of HUVECs treated with or without IL-13 (50 ng/mL) for 20 h, n=5, *P<0.05 vs. control, NS denotes no significant difference. [file Image_5.tif]

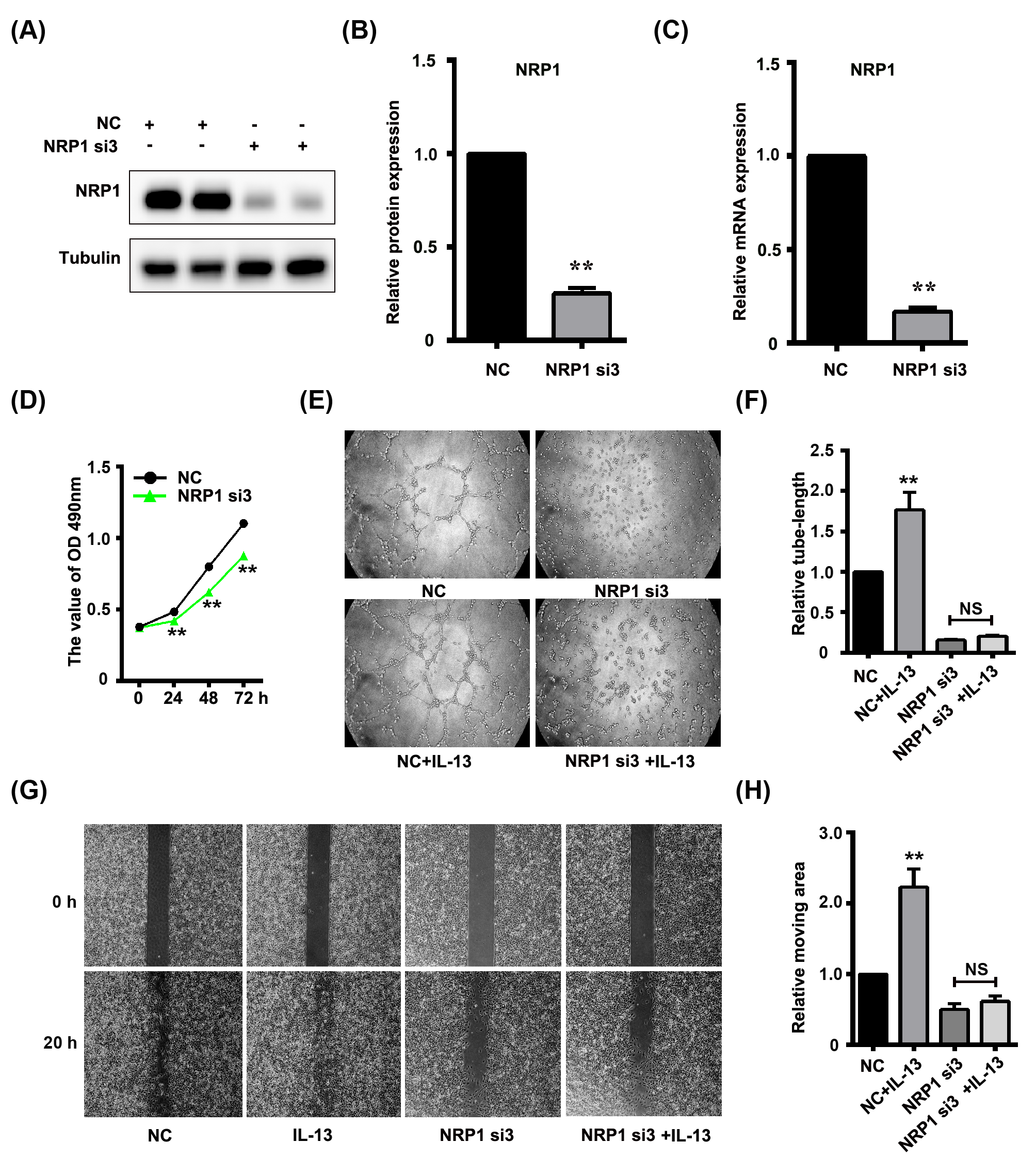

Supplement: Supplementary Figure 6 — Knockdown of NRP1 by NRP1 siRNA 3 inhibits HUVEC migration, proliferation, and tube-formation. (A) The expression of NRP1 was detected by western blotting in HUVECs after transfected with NRP1 siRNA 3 for 48 h. (B) The relative NRP1 expression normalized with tubulin, n=3, *P<0.05 vs. control. (C) The mRNA level of NRP1 was detected by qPCR in HUVECs after transfected with NRP1 siRNA 3 for 48 h, n=3, **P<0.01 vs. control. (D), After transfected with NRP1 siRNA 3 for 48 h, HUVECs were cultured for 0 h, 24 h, 48 h, and 72 h, the cell growth curve of HUVECs were detected by MTT, n=5, **P<0.01 vs. control. (E, F) After transfected with NRP1 siRNA 3 for 48 h, The typical images and relative tube length of HUVECs were detected in the presence or absence of IL-13 (50 ng/mL) n=5, **P<0.01 vs. control, NS denotes no significant difference. (G, H) After transfected with NRP1 siRNA 3 for 48 h, the typical images and the relative moving area of HUVECs treated with or without IL-13 (50 ng/mL) for 20 h, n=5, *P<0.05 vs. control, NS denotes no significant difference. [file Image_6.tif]

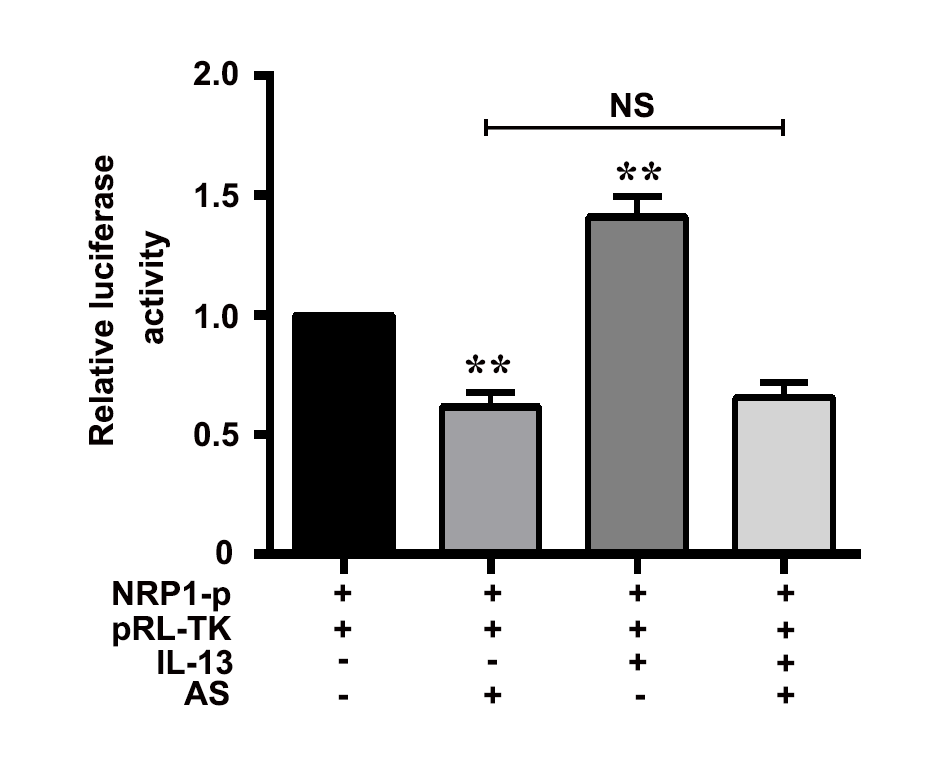

Supplement: Supplementary Figure 7 — Inhibition of STAT6 reduces the promoter activity of NRP1 in the presence of IL-13. Luciferase assay in HUVECs after transfection with pGL3-NRP1 prompter (NRP1-p) and then treated with AS (1 μM), IL-13 (50 ng/mL), and AS combined with IL-13 for 24 h, n=5, **P<0.01 vs. control, NS denotes no significant difference. [file Image_7.tif]

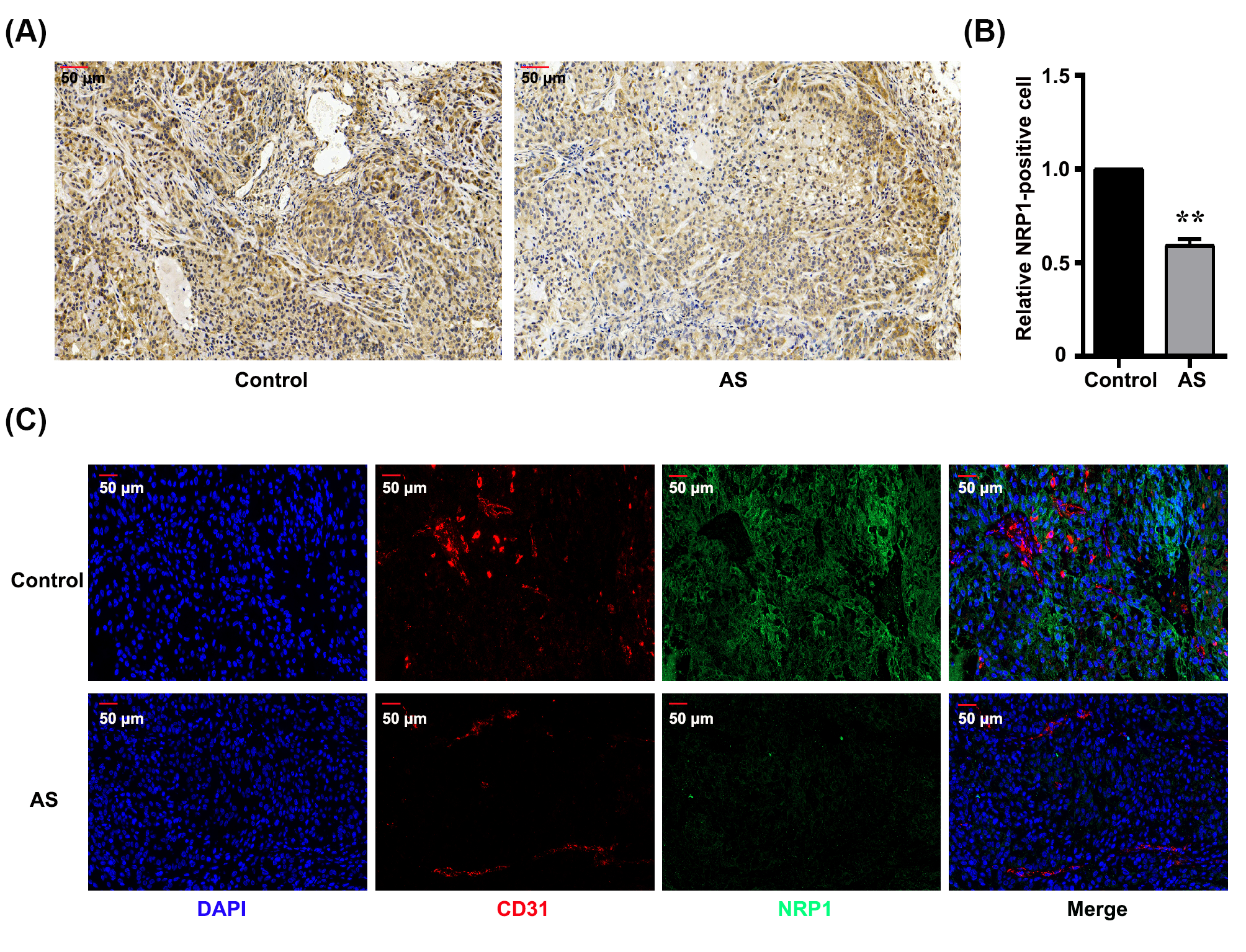

Supplement: Supplementary Figure 8 — Inhibition of STAT6 reduces expression of NRP1 in vivo. (A) The expression of NRP1 in A549 cell-derived tumor treated with or without AS (25 mg/kg body weight) was evaluated by IHC with anti-NRP1 antibody. Scale bar: 50 μm. (B) Relative NRP1 positive cell, n=5, **P<0.01 vs. control. (C) The expression of NRP1 in A549 cell-derived tumor treated with or without AS (25 mg/kg body weight) was evaluated by immunofluorescence with anti-NRP1 antibody (green) and anti-CD31 antibody (red). DAPI was used to display the nucleus. Scale bar: 50 μm. [file Image_8.tif]
